# Statistical methods for elimination of guarantee-time bias in cohort studies: a simulation study

**DOI:** 10.1186/s12874-017-0405-6

**Published:** 2017-08-22

**Authors:** In Sung Cho, Ye Rin Chae, Ji Hyeon Kim, Hae Rin Yoo, Suk Yong Jang, Gyu Ri Kim, Chung Mo Nam

**Affiliations:** 1 Yonsei University, College of Medicine, Seoul, South Korea; 2 Department of Biostatistics and Medical Informatics, Yonsei University College of Medicine, 50-1 Yonsei-ro, Seodaemun-gu, Seoul, 50-1 South Korea; 3 Department of Preventive Medicine, Eulji University College of Medicine, Seoul, South Korea; 4 Department of Biostatistics, Graduate School of Public health, Yonsei University, Seoul, South Korea; 5 Department of Preventive Medicine, Yonsei University College of Medicine, Seoul, South Korea

**Keywords:** Cox regression, Guarantee-time bias, Landmark method, Time-dependent Cox regression

## Abstract

**Background:**

Aspirin has been considered to be beneficial in preventing cardiovascular diseases and cancer. Several pharmaco-epidemiology cohort studies have shown protective effects of aspirin on diseases using various statistical methods, with the Cox regression model being the most commonly used approach. However, there are some inherent limitations to the conventional Cox regression approach such as guarantee-time bias, resulting in an overestimation of the drug effect. To overcome such limitations, alternative approaches, such as the time-dependent Cox model and landmark methods have been proposed. This study aimed to compare the performance of three methods: Cox regression, time-dependent Cox model and landmark method with different landmark times in order to address the problem of guarantee-time bias.

**Methods:**

Through statistical modeling and simulation studies, the performance of the above three methods were assessed in terms of type I error, bias, power, and mean squared error (MSE). In addition, the three statistical approaches were applied to a real data example from the Korean National Health Insurance Database. Effect of cumulative rosiglitazone dose on the risk of hepatocellular carcinoma was used as an example for illustration.

**Results:**

In the simulated data, time-dependent Cox regression outperformed the landmark method in terms of bias and mean squared error but the type I error rates were similar. The results from real-data example showed the same patterns as the simulation findings.

**Conclusions:**

While both time-dependent Cox regression model and landmark analysis are useful in resolving the problem of guarantee-time bias, time-dependent Cox regression is the most appropriate method for analyzing cumulative dose effects in pharmaco-epidemiological studies.

## Background

Extensive studies have elaborated and documented protective effect of aspirin for the primary prevention of cardiovascular diseases, including myocardial infarction (MI), stroke, coronary artery disease (CAD) [1–6]. Distinct from other non-steroidal anti-inflammatory drugs (NSAIDs), aspirin has the capacity to irreversibly inhibit cyclooxygenase (COX) and suppress thromboxane production. Findings of experimental studies have suggested that COX-2 suppression [7–9] or anti-platelet effect of aspirin [10, 11] could interact with cancer cells and play a significant role in blocking tumor angiogenesis, invasiveness and metastatic potential. Accordingly, several observational studies have reported an inverse association between aspirin use and cancer incidence or mortality. However, the results have been inconsistent and controversy still remains regarding its anti-cancer effects [12–15], in part due to variations in study designs and statistical methods for analyzing the effectiveness of aspirin. For example, Fraser et al. [16] conducted a population-based study of breast cancer patients in Scotland, in which Cox’s proportional hazard models was used to assess the relationship between aspirin use and survival. The authors found significantly reduced all-cause (HR = 0.53, 95% CI = 0.45 to 0.63) and breast cancer-specific mortality (HR = 0.42, 95% CI = 0.31 to 0.55) among participants who consumed aspirin following a diagnosis of breast cancer. Similarly, using Cox proportional hazards modeling, Jacobs et al. [17] reported that total cancer incidence is significantly lower among regular long-term aspirin users (RR = 0.84, 95% CI = 0.76 to 0.93). In contrast, a study by McMenamin and colleagues [18] found a non-significant decrease in the risk of lung cancer-specific mortality (*p* = 0.5975) with aspirin use. In this study, the authors used a time-dependent Cox model whereby low-dose aspirin usage was treated as time-varying covariate, with users not considered to be exposed until after a lag of six months following initial prescription. Further, Cardwell et al. [19], in their conditional logistic regression analysis of nested case-control data, also found no evidence for protective effects of low-dose aspirin usage against colon cancer-specific mortality (OR = 1.06; 95% CI = 0.92 to 1.24).

The above-mentioned studies of aspirin use show that when evaluating drug effects, results are often confounded by time-related biases that tend to exaggerate the protective effects, even when the drug has no or little effect [20]. Time-related biases can be further categorized into guarantee-time bias (also known as immortal time bias or time-dependent bias), time-window bias, or time-lag bias. For the purpose of this paper, we focus on guarantee-time bias, which is frequently encountered in time-to-event analyses evaluating drug effects. Guarantee-time bias refers to bias arising from incorrect handling of the time from the beginning of follow-up to the first treatment exposure [21]. The bias occurs if treated patients are assumed to have already been treated from time of cohort entry. For example, in a study of patients with heart disease, heart transplant is a time-varying treatment. While some patients may receive a transplant at the start of follow-up, others may undergo transplant long after the beginning of follow-up or die before undergoing the transplant. Failure to account for time-varying feature of the treatment can result in biased estimation of the treatment effect, in this case, in favor of the treatment group [22].

This study aimed to evaluate the extent of guarantee-time bias in estimation of drug effects on disease outcomes. We conducted simulation studies in which time-fixed, time-dependent Cox model, and landmark analysis were compared for handling guarantee-time bias. For illustrative purposes, effect of cumulative rosiglitazone dose on the risk of hepatocellular carcinoma was investigated using the Korean National Health Insurance Data.

## Method

### Guarantee-Time bias

**Fig. 1:**
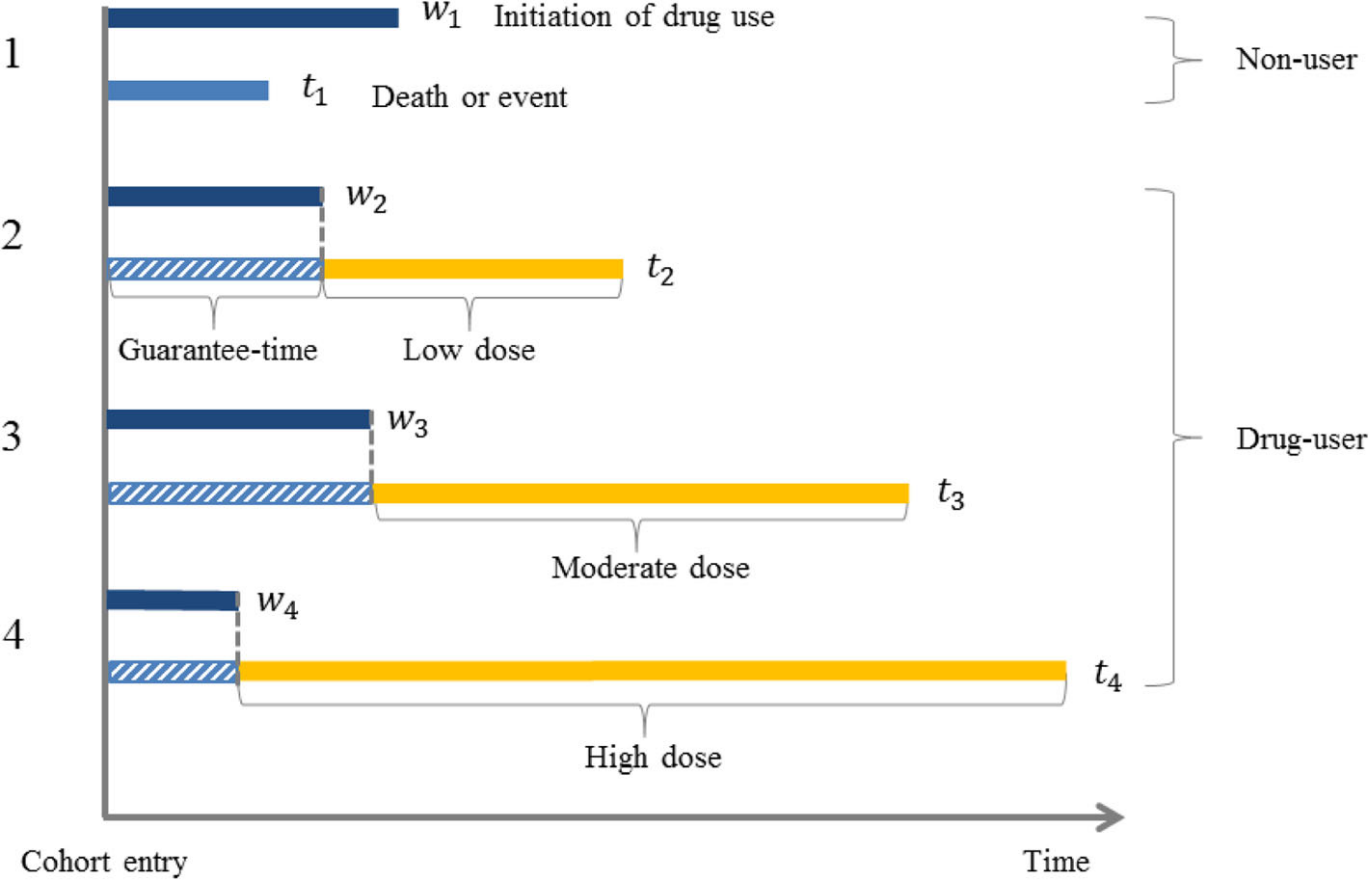
Guarantee-Time Bias Considering four people, time to initiation of drug use ***w***_***i***_ and a time to event ***t***_***i***_ were randomly generated for each person. The person whose value of **T**_0_ is smaller than **W** is allocated to the non-user group (person #1). Otherwise, individuals are allocated to the drug-user group, according to cumulative drug exposure (persons #2 to 4)

Guarantee-time bias in cohort studies can distort the results in favor of the treatment group, depending on the type of event (if it benefits the patient or not) [21, 23, 24]. In detail, consider this study as a cohort study and the situation where drug effect and event occurrence are independent. Event is defined as the disease of interest. Define the random variables *W* and *T*_0_ as the time to initiation of drug usage and time to occurrence of event. The binary random variable *Z* is defined to be *Z* = *I*[*W* ≤ *T*_0_], which represents drug usage. The random variables *T*_0_ and *T*_1_ are defined as the time to event conditional on *Z* = 0 or 1 respectively, i.e. *T* = (1 − *Z*)*T*_0_ + *ZT*_1_. As shown in Fig. 1, the person whose value of *T*_0_ is smaller than W is allocated to the non-user group (person #1). Otherwise, individuals are allocated to the drug-user group, according to cumulative drug exposure (persons #2 to 4). In other words, to have received the treatment implies that the subject survived or was event-free, up to the initiation of drug use. As regards drug-user groups, the longer the survival times, the higher the probability of belonging to the high-dose group. This is the guarantee-time bias phenomenon, i.e. subjects must have lived long enough to receive the treatment and consume large amounts of drug.

### Statistical modeling for Guarantee-Time bias

Our statistical modeling closely follows the paper by Nam and Zelen [25]. For convenience, we consider the situation where the drug usage is binary. The probability density functions of *W* ,  *T*_0_ ,  *T*_1_ will be denoted by *g*(*w*) ,  *q*_0_(*t*) and *q*_1_(*t*) respectively. The survival functions will be denoted by *G*(*w*) = Pr[*W* > *w*] ,  *Q*_0_(*t*) = Pr[*T*_0_ > *t*] and *Q*_1_(*t*) = Pr[*T*_1_ > *t*]*.* Note that by definition *Z* = 1 if the time for drug usage is observed. If the drug usage does not cause a change in the survival function, the conditional density function of non-user group *f*(*t*| *z* = 0) and drug-user group *f*(*t*| *z* = 1) would be the same.

Under the hypothesis that *q*_0_(*t*) = *q*_1_(*t*), if we simply compare the survival functions of the two groups according to the drug usage, the two groups of conditional density functions are not equal to *f*(*t*| *z* = 0) ≠ *f*(*t*| *z* = 1), as it has been proven in the paper by Nam and Zelen [25]. Therefore, it is not appropriate to simply compare the survival functions of two groups according to drug usage.

#### Landmark method

In the landmark method, selected landmark time *τ*_0_ is set, and binary random variable *Z*(*τ*_0_) is defined as *Z*(*τ*_0_) = *I*(*W* < *τ*_0_| *T* > *τ*_0_)*.* The conditional density functions for *Z*(*τ*_0_) can be computed as follows [25]:

$$ {\displaystyle \begin{array}{ll}f\left(t|Z\left({\tau}_0\right)=0\right)=\frac{q_1(t)\left[A(t)-A\left({\tau}_0\right)\right]+G(t){q}_0(t)}{\int_{\tau_0}^{\infty}\left\{{q}_1(x)\left[A(x)-A\left({\tau}_0\right)\right]+G(x){q}_0(x)\right\} dx}\hfill & \mathrm{fo}r\kern0.75em t>{\tau}_0\hfill \\ {}f\left(t|Z\left({\tau}_0\right)=1\right)=\frac{q_1(t)}{Q_1\left({\tau}_0\right)}\hfill & \mathrm{fo}\mathrm{r}\kern1.00em t>{\tau}_0\hfill \end{array}} $$

where *A*(*t*) = 1 − *G*(*t*).

Hence if *q*_0_(*t*) = *q*_1_(*t*), it can be seen that the two functions are the same as

$$ f\left(t|Z\left({\tau}_0\right)=0\right)=f\left(t|Z\left({\tau}_0\right)=1\right)={q}_0(t)/{Q}_0\left({\tau}_0\right)\kern0.5em \mathrm{for}\kern1em t>{\tau}_0. $$

As a result, it is possible to make a valid comparison of two survival functions between the drug-user and non-user groups using the landmark method. In this study, landmark analysis was conducted using time-fixed Cox regression with drug exposure status defined prior to landmark time. One thing to mention in the landmark method is that it is very important to carefully select the landmark time.

#### Time-dependent Cox regression model

In a time-dependent Cox regression model, a time-dependent covariate in the model tracks whether the classifying event has occurred during the estimation process [23]. This method eliminates guarantee-time bias by using drug usage as a time-dependent covariate; subjects are classified as unexposed until the start of drug usage and exposed thereafter [26–28]. The time-dependent Cox regression model has the advantage of using all study follow-up data since it starts analysis at the time of cohort entry. By including all data, this method has increased statistical power over the landmark method [29].

### Simulation setting

We conducted extensive simulations to compare the performance of our three methods, Cox regression, time-dependent Cox regression and landmark method, in terms of empirical type I error and power. We used a discrete-time survival model to describe the effect of cumulative drug dose on the outcome using three indicator variables *Z*_1_(*t*) ,  *Z*_2_(*t*) ,  *Z*_3_(*t*) which represent low, moderate and high-drug dose groups. We set *N* as total sample size and *d* as the number of drug-users. Among *N* subjects, *d* subjects consumed drug for different durations and the dosage was fixed at a constant dose of 0.5 per unit of time. We assumed that drug is taken continuously without interruption until the end of the study or event.

**Fig. 2:**
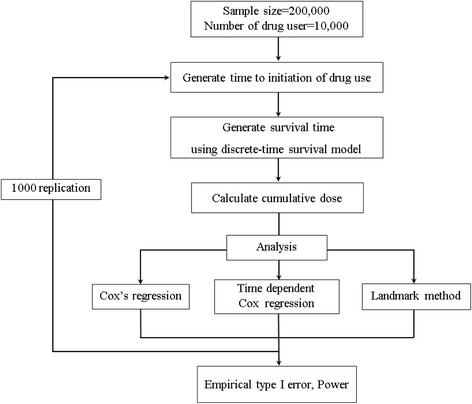
Description of Simulation setting

We divided the total observation period (10-year) into ten intervals as needed for the discrete-time survival model. The overall simulation setting is represented in Fig. 2. We assumed that the drug user rate is 5%. First, we randomly assigned time to initiation of drug use between 0 and 10 for each drug-user. Using these initiation times, we calculated cumulative dose at each time ***t*** and categorized each subject into four groups: non-user group and low(*Z*_1_(*t*) = 1), moderate(*Z*_2_(*t*) = 1), high-dose(*Z*_3_(*t*) = 1) groups. At each discrete time point, the probability of disease occurrence at time t is as follows:

$$ P(t)=\frac{\exp \left({\beta}_0+{\beta}_1{Z}_1(t)+{\beta}_2{Z}_2(t)+{\beta}_3{Z}_3(t)\right)}{1+\exp \left({\beta}_0+{\beta}_1{Z}_1(t)+{\beta}_2{Z}_2(t)+{\beta}_3{Z}_3(t)\right)} $$

If the event occurred within the observation period, survival time is the point of event occurrence. On the other hand, if the event did not occur, survival time is equal to 10 years and censored. For the landmark analysis, we considered subjects whose starting point of drug intake is before landmark time as drug-users, and otherwise non-user. We used 5 and 7 as landmark times, respectively. Subjects who died or who were censored before the landmark time were excluded from the analysis.

Simulations were performed under two different scenarios. In the first scenario, type I error rates for each of the statistical methods were evaluated under the null hypothesis, whereby *β*_1_, *β*_2_ and *β*_3_ values were set to zero to represent a case of no significant protective effect of drug on disease outcome. For the power comparisons in scenario 2, we simulated data from the alternative hypothesis (i.e. cumulative drug dose is associated with disease outcome) for each of the three *β*_0_ values denoting disease incidence: log(0.015), log(5^∗^0.015) , and log(10^∗^0.015). For each simulation setting, 1000 replication datasets were generated. All simulations were performed using the R statistical software [30].

## Results

### Simulation results

The empirical type I errors of three methods under the null hypothesis of no association between cumulative drug dose and disease are illustrated in Table 1. Each value represents the total number of cases out of 1000 replications for which the null hypothesis is incorrectly rejected. As expected, Cox regression model with fixed covariate values tended to produce inflated type I error rates, especially in the moderate and high dose groups. Time-dependent Cox regression and landmark method, on the other hand, displayed well-controlled type I error rates; nearly all values remained close to the nominal level of significance of 0.05, regardless of the degree of disease incidence.

**Table 1:** Estimated Empirical type I error of Cox regression, Time-dependent Cox regression and Landmark methods on simulated data under the null hypothesis that aspirin is not significantly related to cancer

*β*_0_	Cumulative dose	Cox regression	Time-dependent Cox regression	Landmark analyses	
				τ = 5	τ = 7
log(0.015)	Low	214	58	56	57
	Moderate	1000	52	42	45
	High	1000	43	55	47
log(5^∗^0.015)	Low	723	47	56	66
	Moderate	1000	50	40	44
	High	1000	47	46	46
log(10^∗^0.015)	Low	957	52	62	52
	Moderate	1000	59	50	48
	High	1000	46	46	44

Each value represents the number of cases out of 1000 replications for which the null hypothesis is incorrectly rejected

Two values of landmark time (τ) are used, 5 and 7. *β*_0_ values represent disease incidence rate

The power estimates for the alternative hypothesis are displayed in Table 2. Statistical power of the time-dependent Cox regression approach was higher than that of landmark analyses in the high-dose group. In the case of moderate and low-dose groups, the landmark method using landmark time 5 demonstrated higher statistical power compared to the time-dependent Cox regression. This is attributed to the fact that there was extremely small number of cases for the high-dose group, compared with the moderate group.

**Table 2:** Power comparisons of Cox regression, Time-dependent Cox regression and Landmark methods on simulated data under the alternative hypothesis that cumulative drug dose is significantly related to disease outcome

*β*_0_	Cumulative dose	Cox regression	Time-dependent Cox regression	Landmark analyses	
				τ = 5	τ = 7
log(0.015)	Low	214	58	119	74
	Moderate	1000	109	348	181
	High	1000	627	186	396
log(5^∗^0.015)	Low	723	47	289	103
	Moderate	1000	296	792	471
	High	1000	977	452	827
log(10^∗^0.015)	Low	957	52	288	94
	Moderate	1000	322	836	523
	High	1000	989	538	888

*β*_0_ values represent disease incidence rate; τ= Landmark time

Table 3 summarizes the bias and mean squared errors (MSE) of hazard ratios for estimation of the association between cumulative drug dose and outcome. Time-dependent Cox regression had much lower bias than the landmark method and generally showed smaller MSE values compared with the other methods. For instance, for the low-dose group, when the *β*_0_ value was log(0.015), the bias of the time-dependent Cox regression model was 0.0009, while the bias of the landmark analyses were 0.0673 and 0.0416 for *τ* = 5 and *τ* = 7, respectively.

**Table 3:** Bias and MSE of Hazard ratio for estimation of the association between cumulative drug dose and outcome

*β*_0_	Cumulative dose	Cox regression		Time-dependent Cox regression		Landmark analyses			
						τ = 5		τ = 7	
		Bias	MSE	Bias	MSE	Bias	MSE	Bias	MSE
log(0.015)	Low	0.0732	0.0035	0.0009	0.0041	0.0673	0.0062	0.0416	0.0105
	Moderate	0.2735	0.0019	0.0005	0.0043	0.0641	0.0043	0.0469	0.0068
	High	0.6210	0.0004	0.0419	0.0034	0.0437	0.0174	0.0429	0.0061
log(5^∗^0.015)	Low	0.0822	0.0009	0.0007	0.0012	0.0614	0.0020	0.0352	0.0034
	Moderate	0.3002	0.0004	−0.0024	0.0012	0.0585	0.0013	0.0455	0.0023
	High	0.6263	0.0001	0.0392	0.0011	0.0377	0.0048	0.0366	0.0019
log(10^∗^0.015)	Low	0.0944	0.0006	0.0011	0.0008	0.0565	0.0016	0.0300	0.0029
	Moderate	0.3349	0.0002	−0.0032	0.0009	0.0524	0.0011	0.0461	0.0020
	High	0.6331	0.0001	0.0360	0.0009	0.0360	0.0038	0.0338	0.0016

*β*_0_ values represent disease incidence rate, *MSE* Mean squared error, *τ* Landmark time

## Real data example

We applied our simulation findings to a real data from the Korean National Health Insurance Database (NHID). The sample was followed for 12 years from 2002 to 2013. The event of interest was incidence of hepatocellular carcinoma (HCC). Among 47,738 patients with incident diabetes, 203 hepatocellular carcinoma cases were identified. Full details of the study design have been described elsewhere [31, 32]. In the current study, total prescribed doses of rosiglitazone were calculated at each year and summed to produce cumulative doses of follow-up years. Discrete-time survival analysis was used to take into account of the intermittent administration of rosiglitazone and the varying dosage between patients. Based on cumulative dose of rosiglitazone use during the study period, drug users were categorized into low (<1350 mg), moderate (1350–4499 mg), high groups (≥4500 mg). In the landmark analysis, sixth year was chosen as a landmark time since the number of incident HCC was balanced at that time point. Data analysis was carried out using the SAS statistical software version 9.4 [33].

Results based on the NHID data are illustrated in Table 4. In the Cox regression, the risk of HCC incidence was lowest among subjects exposed to high cumulative doses of rosiglitazone (HR = 0.443, 95% CI = 0.218 to 0.899). However, the protective pattern of dose-response relationship and effect sizes were considerably attenuated in the time-dependent Cox regression and landmark analysis.

**Table 4:** Results of the effect of rosiglitazone on hepatocellular carcinoma based on real data from the Korean National Health Insurance Database

Cumulative dose	Cox regression				Time-dependent Cox regression				Landmark analyses			
	HR	95% CI		*p*-value	HR	95% CI		*p*-value	HR	95% CI		*p*-value
Low	0.852	0.420	1.731	0.659	1.155	0.568	2.347	0.691	1.059	0.332	3.374	0.923
Moderate	0.710	0.315	1.602	0.410	0.983	0.435	2.220	0.967	0.775	0.189	3.167	0.722
High	0.443	0.218	0.899	0.024	0.778	0.381	1.587	0.490	0.812	0.295	2.233	0.687

*HR* Hazard ratio, *95% CI* 95% Confidence interval

## Discussion

In this study, we examined the phenomenon of guarantee-time bias through statistical modeling and simulation study. Specifically, our simulation study assessed the performance of three methods, namely Cox regression, time-dependent Cox regression and landmark method based on time-fixed Cox regression. These methods depend upon the proportional hazard assumption. According to our simulation results, time-fixed Cox regression was shown to be vulnerable to guarantee-time bias [20, 22]. Pharmaco-epidemiological studies typically involve the use of time-varying exposures, such as cumulative dose. Hence, under such situations, applying the time-fixed Cox regression approach can induce bias due to model misspecification. Our results are in accordance with results previously reported by Mi et al. [29]. However, unlike previous studies, we performed a simulation by including cumulative dose groups as exposures.

Landmark analysis has been suggested in previous studies as a simplified alternative to time-dependent Cox regression for elimination of guarantee-time bias in time-to-event data. In our simulation, landmark method was comparable to the time-dependent Cox regression method in terms of type I error. But landmark analysis tended to slightly increase MSE. One possible explanation for these results could be the sample size. For landmark analysis to produce efficient estimates, it is imperative that optimal landmark time is specified. As illustrated by our results, if the landmark point is too early, there is a greater possibility of imbalanced observations among treatment groups. On the other hand, if the landmark point is too late, a significant proportion of events may be omitted, giving rise to insufficient number of cases to achieve adequate power. Thus, it is important that landmark studies are designed with sufficient number of participants to maintain adequate statistical power. Additionally, the landmark method will produce estimates with minimal bias conditional upon the treatment being evenly distributed across the follow-up [29]. Recently, the use of landmark super models, a pooled summary analysis of several landmarks, has been advocated to remedy the problem of low statistical power related to the landmark method [34, 35]. Further studies are warranted to compare the performance of landmark super models with the time-dependent Cox model.

Nevertheless, the landmark approach has several advantages over the time-dependent Cox regression model. Most notably, this method has the advantage of computational simplicity because drug use is defined as a time-fixed covariate by using landmark time. Thus, one can visualize the survival curve of drug users using the Kaplan-Meier method. But, unconditional Kaplan-Meier estimates of time-varying status of drug users are unstable because the number of drug users can be quite small at early time point [36].

## Conclusions

In conclusion, to avoid guarantee-time bias in observational studies of drug effects, we recommend incorporating time-dependent exposure status in the analysis. While both time-dependent Cox regression model and landmark analysis were found to be useful in resolving the problem of guarantee-time bias, time-dependent Cox regression was the most appropriate method for analyzing cumulative and long-term drug exposure. We recommend the time-dependent Cox regression for estimating hazard ratios of cumulative doses. Alternatively, due to its graphical capabilities, the landmark method may be a suitable alternative for visualizing survival curves for treatment groups.

## Abbreviations

HCC: hepatocellular carcinoma
MSE: Mean squared error
NHID: National Health Insurance Database
OR: Odds ratio
